# Integrated analysis of the prognostic and oncogenic roles of OPN3 in human cancers

**DOI:** 10.1186/s12885-022-09219-7

**Published:** 2022-02-18

**Authors:** Wei Zhang, Jianglong Feng, Wen Zeng, Zhi He, Wenxiu Yang, Hongguang Lu

**Affiliations:** 1 Department of Dermatology, Affiliated Hospital of Guizhou Medical University, No.28 Guiyi Road, Guiyang, Guizhou 550001 P.R. China; 2 Department of Pathology, Affiliated Hospital of Guizhou Medical University, Guiyang, Guizhou China

**Keywords:** OPN3, Pancancer, Prognosis, C7orf70, C7orf25, Ribosome

## Abstract

**Background:**

Emerging cell- or tissue-based evidence has demonstrated that opsin 3 (OPN3) mediates a variety of pathological processes affecting tumorigenesis, clinical prognosis, and treatment resistance in some cancers. However, a comprehensive analysis of OPN3 across human cancers is unavailable. Therefore, a pancancer analysis of OPN3 expression was performed and its potential oncogenic roles were explored.

**Methods:**

The expression and characterization of OPN3 were evaluated among 33 tumour types using The Cancer Genome Atlas (TCGA) dataset. Additionally, the OPN3 RNA level and overall survival (OS) in relation to its expression level in 33 cancer types were estimated. Based on the analysis above, 347 samples from 5 types of tumours were collected and detected for the protein expression of OPN3 by immunohistochemical assay. Furthermore, the biological role of OPN3 in cancers was evaluated via gene set enrichment analysis (GSEA).

**Results:**

The OPN3 expression level was heterogeneous across cancers, yet a remarkable difference existed between OPN3 expression and patient overall survival among the 7 types of these 33 cancers. Consistently, a high immunohistochemical score of OPN3 was significantly associated with a poor prognosis among patients with 5 types of tumours. Additionally, OPN3 expression was involved in cancer-associated fibroblast infiltration in 5 types of tumours, and promoter hypomethylation of OPN3 was observed in 3 tumour types. Additionally, OPN3 protein phosphorylation sites of Tyr140 and Ser380 were identified via posttranscriptional modification analysis, suggesting the potential function of Tyr140 and Ser380 phosphorylation in tumorigenesis. Furthermore, the enrichment analysis was mainly concentrated in C7orf70, C7orf25 and the “ribosome” pathway by GSEA in 5 types of cancers, indicating that OPN3 might affect tumorigenesis and progression by regulating gene expression and ribosome biogenesis.

**Conclusions:**

High expression of OPN3 was significantly associated with a poor clinical prognosis in five types of cancers. Its molecular function was closely associated with the ribosomal pathway.

**Supplementary Information:**

The online version contains supplementary material available at 10.1186/s12885-022-09219-7.

## Introduction

Opsins, a large family of cell surface photoreceptors, were first described in the eye and play multiple roles in phototransduction in the visual process [1]. However, some opsins not only serve light-dependent functions but also play light-independent roles, especially in extraocular tissues. Opsin 3 (OPN3), also known as encephalopsin, was first identified as an extraocular opsin [2], which has been demonstrated to be associated with light-independent functions such as the regulation of melanogenesis and apoptosis in epidermal melanocytes [3, 4]. Notably, it has been found that functional links between OPN3 and tumorigenesis of lung cancer, skin melanoma and clinical prognosis [5–7]. For lung cancers, overexpression of OPN3 was shown to promote epithelial-mesenchymal transition and metastasis in lung adenocarcinoma [5]. OPN3 was also upregulated among patients with postoperative recurrence of pulmonary carcinoid tumours [6]. Recently, a study found that high expression of OPN3 was involved in the metastatic phenotype and a poor prognosis in acral lentiginous melanoma [7]. Moreover, a previous study revealed that OPN3 was associated with 5-fluorouracil resistance in hepatocellular carcinoma cells, as its depletion activated the antiapoptotic pathway and ultimately influenced hepatocellular carcinoma sensitivity to chemotherapy [8]. In addition, OPN3 can mediate blue light-emitting diodes to induce autophagy in human colon cancer cells and suppress cell growth [9]. Collectively, previous findings demonstrated that OPN3 plays multiple important roles in tumorigenesis, clinical prognosis, and treatment resistance in various cancers. However, the expression and function of OPN3, which is widely expressed in multiple tissues, remain unknown in human cancers.

Pancancer analysis is able to examine the genes whose mutation is conducive to oncogenesis, as well as the expression of the similarities and differences between different cancers [10]. Thus, it is important for pancancer analysis to assess the association with clinicopathological features and prognosis and to explore potential molecular functions. Pancancer analysis was realized after the birth of some tumour databases, such as The Cancer Genome Atlas (TCGA) [10]. In this study, the expression and characterization of OPN3 in different human cancers, as well as its association with clinical prognosis and potential functional roles was the focus. Its gene expression level and survival analysis were first evaluated among 33 tumour types by TCGA data, and further OPN3 aberrations were analysed across tumour types. Furthermore, the expression of OPN3 was performed to verify the association between OPN3 expression level and clinical prognosis by immunohistochemical staining in cancer tissues, in which there was a significant difference between OS and different OPN3 expression levels from the TCGA dataset. Finally, the molecular mechanism of OPN3 was investigated in the TCGA dataset using the gene set enrichment analysis (GSEA) method.

## Materials and methods

### Data collection

The gene expression data and related clinical overall survival information for 33 tumour types were collected from TCGA datasets (https://portal.gdc.cancer.gov/). In addition, the Chinese Glioma Genome Atlas (CGGA) dataset (http://www.cgga.org.cn/index.jsp) and Database of Hepatocellular Carcinoma Expression Atlas (HCCDB, http://lifeome.net/database/hccdb/home.html) were used to validate the expression and characterization of OPN3 in glioma and hepatocellular carcinoma, respectively [11, 12]. Our cohort was composed of 5 types of tumours from the Affiliated Hospital of Guizhou Medical University. Haematoxylin and eosin (H&E)-stained sections were reviewed and evaluated, and samples fulfilling criteria for the appropriate diagnoses of various cancers were selected for study. Archived formalin-fixed paraffin-embedded (FFPE) blocks were cut to make 4 μm sections for immunohistochemistry (IHC) staining. The study was approved by the Ethics Committees of Affiliated Hospital of Guizhou Medical University.

### OPN3 gene expression and survival analysis

OPN3 gene expression in the 33 kinds of cancers from TCGA data was analysed using the Gene Expression Profiling Interactive Analysis (GEPIA) browser (http://gepia.cancer-pku.cn/) [13], and TIMER (http://timer.comp-genomics.org/) [14]. Kaplan–Meier (KM) survival curves combined with a log-rank test were used to test the differences in prognosis between the high- and low-expression OPN3 groups (according to the median expression value of OPN3) using the survival R package [15]. OPN3 gene differential expression and overall survival analyses in the glioma from CGGA dataset were analysed using the Kaplan–Meier plotter online tools of CGGA (http://www.cgga.org.cn/analyse/RNA-data.jsp). Additionally, the pancancer analysis of OPN3 variations and DNA methylation profiles were assessed by the cBio Cancer Genomics Portal tool (http://cbioportal.org) [16] and UALCAN (http://ualcan.path.uab.edu/) [17], respectively. TIMER was also used for the analysis of tumour-infiltrating immune cells, including cancer-associated fibroblasts [18].

### Gene set enrichment analysis

Gene Ontology molecular function (GO_MF) and Kyoto Encyclopedia of Genes and Genomes (KEGG) analyses of TCGA data were conducted using the LinkedOmics database platform (http://www.linkedomics.org/login.php) [19, 20]. GO terms and KEGG pathways with *P* < 0.05 and FDR < 0.25 were considered remarkably enriched.

### IHC analyses of OPN3 expression

Details about the methods and further the semiquantitative assessment followed previous reports [7]. Briefly, 4 μm sections with different types of tumour tissues were dewaxed and rehydrated according to standard methods. Antigen retrieval was conducted with retrieval solution (ethylenediaminetetraacetic acid [EDTA], pH 9.0, ZLI-9069 from ZSGB-BIO, Beijing, China) for 4 min using a pressure cooker. H_2_O_2_ (PV-9000; ZSGB-BIO) was applied to block endogenous enzyme activity, and the samples were subsequently incubated in a serum-free blocking solution (ZLI-9056; ZSGB-BIO). Then, the primary antibody against OPN3 (MD4034-100; Medical Discovery Leader (MDL), Beijing, China) was diluted 1:300 at 4 °C overnight, followed by treatment with the UltraView Polymer DAB Detection Kit (Ventana/Roche) according to the recommended manufacturing protocol.

OPN3 expression on all stained slides was scored by two independent investigators. The semiquantitative assessment method was conducted by using percentages of 3 + (strong), 2 + (moderate), 1 + (weak), and 0 (negative) staining of tumour cells for each sample. The overall score was calculated by the percentage of positive tumour cells (3 × x % + 2× x % + 1 × x % = total score) to equal a range of 0-300 [21].

### Statistical analyses

R version 3.6.1 and GraphPad Prism (version 8.0) software were used for statistical analysis. Continuous variables are presented as the mean ± SD or median with interquartile range (IQR) when distribution was skewed. The analysis of variance to compare means of two or more than two groups was performed by *t* tests or one-way ANOVA with Tukey’s post-test analysis of variance. The Mann–Whitney (two groups) test was used to compare the nonparametric distributions. Survival analyses were conducted via the Kaplan–Meier method. A univariate Cox regression model was applied to assess adjusted hazard ratios (HRs) and 95% confidence intervals (CIs) for outcomes. Statistically significant differences were considered when *P* < 0.05 (***P < 0.001, **P < 0.01, * *P* < 0.05).

## Results

### Pancancer analysis of OPN3 expression and survival analysis in various cancers

**Fig. 1:**
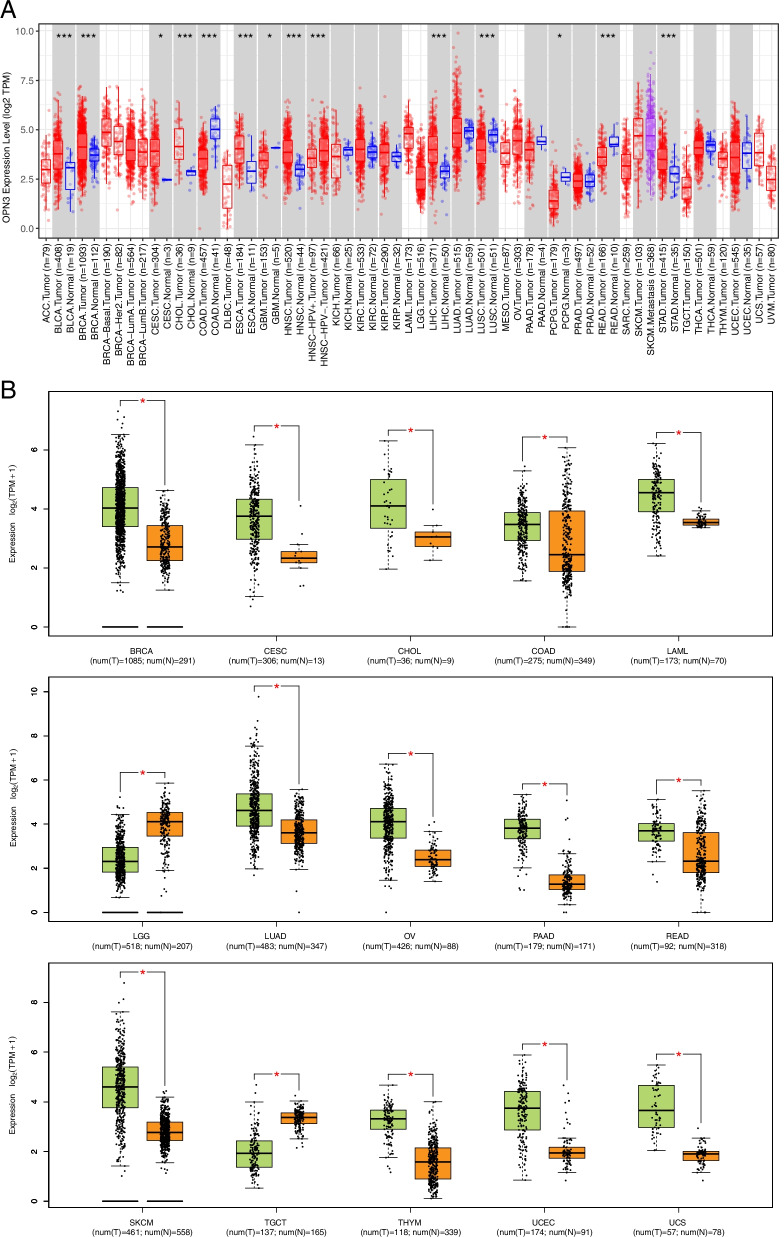
Gene expression of OPN3 in different tumour types or specific cancer subtypes. **A** In the TCGA project, the expression status of OPN3 in 33 subtypes of cancers * *P* < 0.05; ** *P* < 0.01; *** *P* < 0.001. **B** The expression difference of OPN3 in various cancers combined TCGA dataset with GTEx dataset. Log2 (TPM + 1) was used for log-scale. * *P* < 0.05

The differential expression of OPN3 gene in 33 cancer types was compared using TCGA data, which found that the TPM (Trans Per Million) value of OPN3 RNA level was higher in 8 types of cancers including BLCA (Bladder Urothelial Carcinoma), BRCA (Breast invasive carcinoma), CESC (Cervical squamous cell carcinoma and endocervical adenocarcinoma), CHOL (Cholangiocarcinoma), ESCA (Oesophageal carcinoma), HNSC (Head and Neck squamous cell carcinoma), LIHC (Liver hepatocellular carcinoma), STAD (Stomach adenocarcinoma), compared with adjacent normal tissues (Fig. 1A), whereas downregulating in those cancers of COAD (Colon adenocarcinoma), GBM (Glioblastoma multiforme), LUSC (Lung squamous cell carcinoma), PCPG (Pheochromocytoma and Paraganglioma), READ (Rectum adenocarcinoma) (Fig. 1A). After adding the normal tissue in the GTEx (Genotype-Tissue Expression) dataset as controls, the expression difference of OPN3 was assessed between the normal tissues and cancer tissues. As presented in Fig. 1B, OPN3 had significantly high expression in most cancer types, including BRCA, COAD, LAML (acute myeloid leukaemia), OV (ovarian serous cystadenocarcinoma), PAAD (pancreatic adenocarcinoma), READ (rectum adenocarcinoma), THYM (thymoma), UCEC (uterine corpus endometrial carcinoma), CESC, LUAD (lung adenocarcinoma), SKCM (skin cutaneous melanoma) and UCS (uterine carcinosarcoma), while OPN3 was expressed at low levels in LGG (brain lower grade glioma) and TGCT (testicular germ cell tumours). Collectively, these data suggest that the expression of OPN3 at the RNA level was heterogeneous across human cancers.

**Fig. 2:**
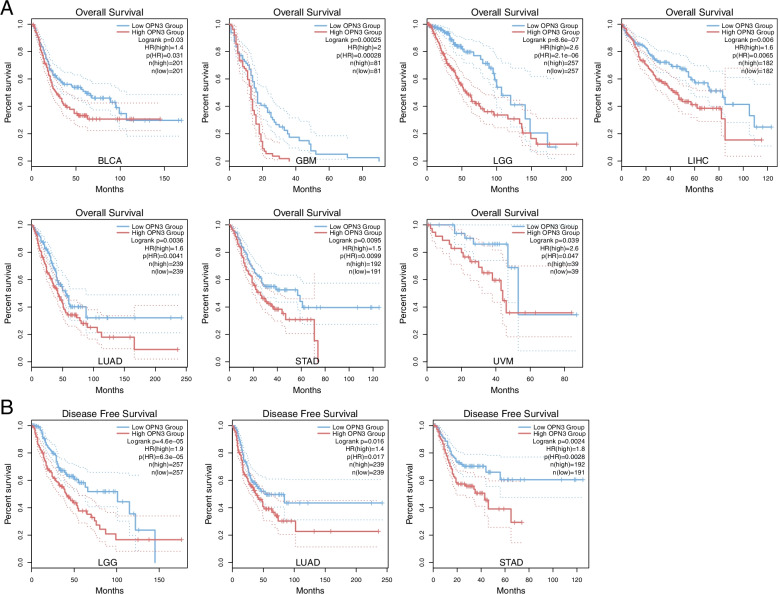
Survival analysis of 7 types of cancer patients between low and high OPN3 expression groups according to OPN3 expression of median value using the Kaplan–Meier method. **A** Overall survival (OS) curve between patients with high and low expression of OPN3 in the TCGA dataset. **B** Disease Free Survival (DFS) curve between patients with high and low OPN3 expression in different tumours

Next, using the TCGA project, the associations between OPN3 expression and the survival status of the 33 tumour types were estimated by log-rank tests. In seven types of cancers, including BLCA, GBM, LGG, LIHC, LUAD, STAD and UVM, Kaplan–Meier survival analysis showed that a significant difference in patient overall survival was found between the low and high OPN3 expression groups according to the OPN3 expression median value (Fig. 2A), revealing that high OPN3 expression was associated with shorter overall survival. In addition, the effects of OPN3 on disease-free survival (DFS) were also tested in seven types of cancers. It was found that the OPN3 expression level markedly affected the survival index of DFS in LGG, LUAD and STAD patients (Fig. 2B), indicating that high OPN3 expression was associated with poor survival. Considering that OPN3 expression and its association with clinicopathological features and prognosis in lung cancer and melanoma have been reported [5, 7], in the next section, the characteristics of OPN3 among the other five cancer types are the primary focus.

### Validation of the OPN3 expression signature in five cancer types

**Fig. 3:**
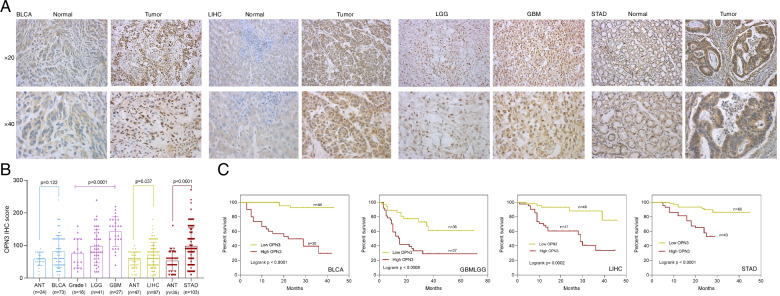
Expression difference of OPN3 protein in 5 tumour types. **A** OPN3 expression in representative cancer cases from 5 tumour types via immunohistochemistry (IHC) staining (× 20, × 40 magnification; Normal: adjacent normal tissues). **B** The IHC staining score of OPN3 differs significantly between tumour tissues and adjacent normal tissues (ANTs) or different grades. **C** Overall survival analysis of tumour patients with different IHC scores of OPN3 (low OPN3 vs. high OPN3) based on the median expression value

To determine the expression signature of OPN3 at the protein level, immunohistochemistry (IHC) staining of the above five types of cancers (BLCA, GBM, LGG, LIHC and STAD) was conducted (Fig. 3). The results showed that the protein level of OPN3 was higher in LIHC and STAD tumours than in adjacent normal tissues (Fig. 3A-B), consistent with its RNA expression level, whereas OPN3 scores of BLCA were not significantly different between tumour and adjacent normal tissues. In terms of glioma, the difference among different grades (I- IV; LGG: grade II-III, GBM: grade IV) due to a lack of adjacent normal tissues was compared. In contrast to grade I glioma and LGG, OPN3 was expressed at a higher level in GBM (*p* < 0.0001 and *p* = 0.001, respectively) (Fig. 3A-B). Similar to the results from TCGA dataset, in the samples, based on OPN3 score of median value, prognostic analysis was made between patients with high and low expression of OPN3 via the Kaplan–Meier method, which showed that high IHC score of OPN3 was associated with worse overall survival in these cancer types (Fig. 3C). Together, these results suggested that the upregulation of OPN3 expression was associated with poor disease outcome in five types of cancers.

Next, Cox regression analysis of prognostic factors for OS of BLCA, GBMLGG, LIHC and STAD patients was performed (HR = 13.03 [95% CI: 3.76-45.17], *p* < 0.001; HR = 3.15 [95% CI: 1.54-6.43], *p* = 0.002; HR = 5.26 [95% CI: 1.97-14.04], p = 0.001; HR = 5.05 [95% CI: 2.06-12.38], *p* < 0.001, respectively) (Fig. S1), which showed that high OPN3 expression was significantly related to worse overall survival in these cancer types. Thus, these promising findings indicated that OPN3 may be a potential indicator for the assessment of cancer prognosis.

### Association between OPN3 and clinicopathologic variables of glioma

As we showed above, at the glioma RNA level, OPN3 appeared to be downregulated in LGG and GBM compared to normal tissues. Paradoxically, the overexpression of OPN3 was associated with a poor prognosis in LGG and GBM. Additionally, the verification of OPN3 protein levels was not able to fulfil the lack of normal tissues as controls in glioma. Therefore, the gene expression difference of OPN3 was compared between gliomas of different grades using the CGGA dataset (Fig. S2). In contrast to LGG, the OPN3 gene was expressed at a higher level in GBM, which was consistent with the expression trend of OPN3 protein levels increasing gradually from grade II (LGG) to IV (GBM) glioma. In addition, OPN3 expression in grade II-IV gliomas with IDH mutation or 1p19q deletion was lower in the CGGA dataset than in IDH wild-type gliomas (*p* < 0.005). The results of survival analysis in glioma from the CGGA dataset were consistent with those in the TCGA dataset (Fig. S2). Together, these results suggested that the upregulation of OPN3 expression was associated with clinicopathological features and poor disease outcome of glioma. Additionally, we confirmed OPN3 expression in LIHC using the HCCDB dataset. The results showed that tumours, in contrast to adjacent normal tissues, had higher RNA levels of OPN3 expression in nine out of the ten HCCDB datasets (Fig. S3).

### Pancancer analysis of OPN3 genetic alteration

**Fig. 4:**
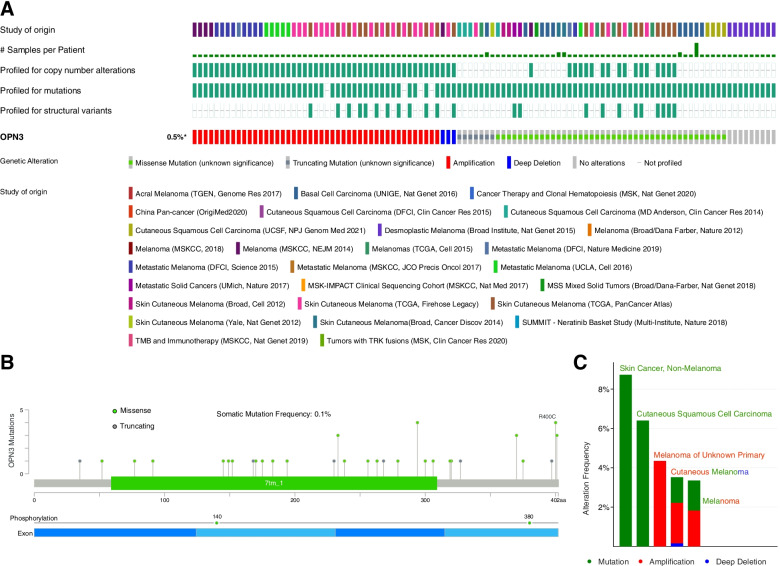
Variation analysis of the OPN3 gene in different pancancer datasets from the cBioPortal database. **A** The types of OPN3 genetic alterations in different pancancer datasets. **B** Missense mutation and phosphorylation modification of OPN3 in the pancancer analysis. **C** Tumour types of OPN3 variants in two different pancancer datasets

Furthermore, OPN3 gene alterations were analysed in 8 different pancancer and 19 skin cancer datasets from the cBioPortal database [16]. It was found that the frequency of gene mutations, including missense mutations, truncating mutations, amplifications and deep deletions, was 0.5% (Fig. 4A-B) and mainly occurred in cutaneous squamous cell carcinoma, basal cell carcinoma and melanoma (Fig. 4C). As shown in Fig. 3B, the percentage of these samples with a somatic mutation in OPN3 was 0.1%. Interestingly, OPN3 protein phosphorylation sites of Tyr140 and Ser380 were identified via posttranscriptional modification analysis (Fig. 4B), suggesting the potential function of Tyr140 and Ser380 phosphorylation in tumorigenesis.

Additionally, OPN3 DNA methylation levels in five types of tumours and normal tissues were assessed using UALCAN [17]. In the TCGA cohort, a significantly reduced methylation level at the promoter region of OPN3 was observed in 3 types of tumours, including BLCA, LIHC, and LUAD, comparable to normal tissues. These results were consistent with the expression level of OPN3 between tumour and normal tissues, as shown in Fig. S4.

**Fig. 5:**
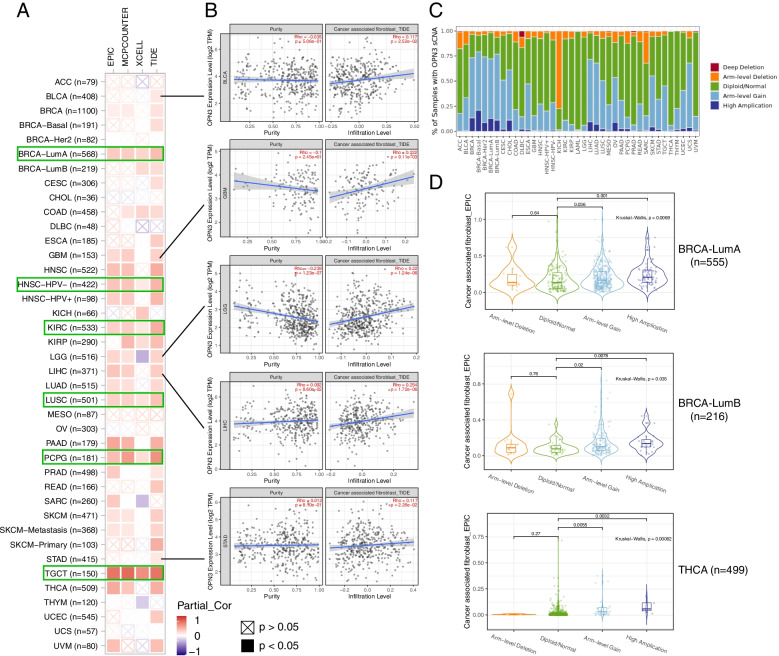
Correlation analysis between OPN3 expression level and infiltration of cancer-associated fibroblasts (CAFs) across all types of cancer in TCGA (**A)** using different algorithms (EPIC, MCPCOUNTER, XCELL, TIDE) [22] and (**B)** in five types of cancer by the TIDE algorithm. The green box indicates that there is a positive correlation in all the algorithms. **C** The features of somatic copy number alterations (sCNAs) of OPN3 across all types of cancer in the TCGA dataset. **D** The significant difference in CAF infiltration level in different statuses of OPN3 sCNAs in BRCA-luminal A (lumA), BRCA-luminal B (lumB) and THCA based on the EPIC algorithm

Additionally, immune infiltration of the cancer microenvironment was evaluated in diverse cancer types of TCGA [18]. An significant positive correlation between most cancers and the infiltration value of cancer-associated fibroblasts was observed (Fig. 5A), especially in TGCT (testicular germ cell tumours), PCPG (pheochromocytoma and paraganglioma), BRCA (breast invasive carcinoma), KIRC (kidney renal clear cell carcinoma), and LUSC (lung squamous cell carcinoma). According to the quanTIseq algorithm [23], correlation analysis revealed that OPN3 was positively correlated with cancer-associated fibroblasts in the above five cancer types (Fig. 5B). Additionally, the cancer-associated fibroblasts between different somatic copy number alterations (sCNAs) of OPN3 were assessed, including “deep deletion”, “arm-level deletion”, “diploid/normal”, “arm-level gain”, and “high amplification” (Fig. 5C-D). The “arm-level gain” and “high amplification” of OPN3 in BRCA-luminal A (lumA), BRCA-luminal B (lumB) and THCA (thyroid carcinoma) were significantly associated with the infiltration value of cancer-associated fibroblasts (*p* < 0.05) based on the EPIC algorithm [22].

### Gene Set Enrichment Analysis (GSEA) of OPN3 in five types of cancers

**Fig. 6:**
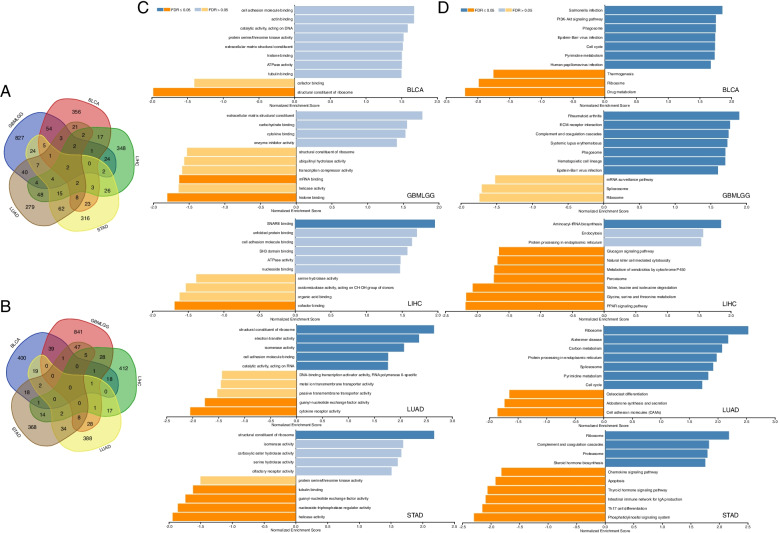
Gene set enrichment analysis of OPN3 in five types of cancer. The intersection of OPN3 and the top 500 OPN3-associated genes (**A**, positive correlation of genes; **B**, negative correlation of genes) in each cancer type. The GO (**C**) and KEGG (**D**) pathways that were enriched by the top ranked genes in the low and high OPN3 expression groups were detected by GSEA. For each analysis, the number of gene set permutations was set to 1000. The nominal (NOM) *P* value, false discovery rate (FDR < 0.25) and normalized enrichment score (NES) were used to identify the pathways enriched in each phenotype

To further investigate the potential molecular mechanism of OPN3 in 5 types of cancers (BLCA, GBMLGG, LIHC, LUAD, STAD), TCGA mRNA-seq data was first measured by Pearson’s correlation analysis between OPN3 and its coexpressed genes. The intersection of OPN3 and the top 500 OPN3-associated genes from the most related modules showed 2 genes (C7orf70 and C7orf25) closely related to the upregulation of OPN3 expression in all 5 types of cancers (Fig. 6A). Additionally, GSEA was performed between samples with low and high OPN3 expression to identify OPN3-related signalling pathways using GO and KEGG pathway enrichment analyses. As shown in Fig. 6, the notably dysregulated terms were primarily enriched in “structural constituent of ribosome”, “ribosome”, partly involved in “spliceosome”, “phagosome”, and “cell cycle”. Thus, these results may provide insights into the cellular biological effects of OPN3, which could regulate the ribosome pathway in tumours and further affect tumorigenesis and progression.

## Discussion

Since it is widely expressed in a variety of human tissues, such as the brain, retina, skin, liver, heart, lung and pancreas, OPN3 is also known as panopsin [24, 25]. Interestingly, OPN3, belonging to the photosensitive opsin family, was unexpectedly expressed in some nonphotosensitive tissues under physiological conditions. Recently, its light-independent function has been of interest in human extraocular tissues. For instance, in human epidermal melanocytes, OPN3 can act as a negative regulator of melanogenesis in a light-independent way by modulating melanocortin 1 receptor signalling [3]. Our group demonstrated that without light illumination, downregulation of OPN3 induces apoptosis of melanocytes through the mitochondrial apoptotic pathway [4]. TGFβ2 is able to upregulate the tyrosinase activity of melanocytes through the light-independent function of OPN3 in a TGFβ2 receptor-independent manner [26]. In human tumours, previous studies showed that the OPN3 gene was upregulated in lung adenocarcinoma and skin melanoma, which was associated with the metastatic phenotype and unfavourable prognosis [5–7]. Additionally, another light-independent function of OPN3 was that its depletion triggered 5-fluorouracil resistance in liver cancer cells via the antiapoptotic pathway [8]. Altogether, OPN3 is associated with tumorigenesis and progression; however, the expression and role of OPN3 in other tumours remain unclear.

In this study, we started with a pancancer analysis of OPN3 opsin expression using the TCGA dataset. Then, we validated the OPN3 expression signature in several tumours by our cohort and other CGGA and HCCDB databases. These results revealed that OPN3 expression is heterogeneous across multiple tumours, which suggested that abnormal expression of OPN3 may play a role in oncogenesis. In particular, we found that high expression of OPN3 in 7 types of cancer tissues was remarkably associated with poor prognosis. Therefore, upregulation of OPN3 may contribute to carcinogenesis in human cancers, especially in the seven cancer types. Furthermore, the analysis of OPN3 gene alterations showed that the frequency of gene mutations was only 0.5% and mainly occurred in cutaneous squamous cell carcinoma, basal cell carcinoma and melanoma. The percentage of these samples with a somatic mutation in OPN3 was only 0.1%, which indicated that OPN3 might not be a driver gene of initial tumorigenesis but could play a vital role in promoting tumorigenesis and progression. Moreover, OPN3 protein phosphorylation sites of Tyr140 and Ser380 were identified via posttranscriptional modification analysis. Interestingly, previous reports indicated that the C-terminus of OPN3, containing 13 potential sites for serine/threonine phosphorylation, may be correlated with arrestin-mediated receptor internalization [27, 28] and may further result in sustained signalling [29]. Thus, it is suggested that the potential function of Tyr140 and Ser380 phosphorylation may be associated with tumorigenesis. However, this observation merits further molecular assays for further exploration of the potential role of two phosphorylation sites. In addition, a significant positive correlation between most cancers and the infiltration value of cancer-associated fibroblasts was observed. Cancer-associated fibroblasts, as prominent components of the tumour microenvironment, are closely linked to the initiation, progression or metastasis of cancer [22, 23]. Thus, OPN3 was closely related to enhancing cancer-associated fibroblasts that participate in modulating the function of various tumour-infiltrating immune cells [24, 25].

To our knowledge, however, the molecular function of OPN3 in cancer has not yet been reported. Therefore, we conducted GSEA to study the molecular mechanisms of OPN3 in carcinogenesis and progression, which demonstrated that OPN3 in 5 types of cancers remarkably correlates with modules of C7orf70 and C7orf25 and the “Ribosome” pathway. The C7orf70 gene, also called STAT3 interacting protein as a repressor (SIPAR), is composed of 2 exons on human chromosome 7p22.1, which encodes a 259 amino acid protein [30]. A previous study found that SIPAR promotes the dephosphorylation of STAT3 and further affects the progression of melanoma through physical interaction with STAT3 [31]. C7orf25 (chromosome 7 open reading frame 25) encodes 12 proteins, but most of these proteins have an unknown function. One of these is UPF0415, which may be associated with ATP-dependent protein breakdown in the proteasome pathway and protein activation [32, 33]. Recent studies suggested that the abnormal expression of enhancer-associated C7orf25 was involved in unfavourable prognosis of GBM, RNA metabolism and gene expression [34, 35]. The ribosome is an essential component of the protein translation machinery, and dysregulation of its biogenesis (upregulation of biogenesis and defection of biosynthesis) may lead to cancer development [36, 37]. Thus, OPN3 may affect carcinogenesis and progression by regulating the ribosomal pathway in cancer.

Taken together, these features suggest that OPN3 is involved in a poor prognosis in some types of cancers, including BLCA, GBM, LGG, LIHC, LUAD, STAD and UVM, mainly by OPN3 gene variations, epigenetic modification (methylation and phosphorylation) patterns and/or affecting the infiltration of cancer-associated fibroblasts. However, additional work is required to evaluate the molecular mechanism of OPN3 as a promotor in tumorigenesis and progression.

## Conclusions

In conclusion, we demonstrated that the high expression of OPN3 was associated with a poor prognosis in BLCA, GBM, LGG, LIHC, LUAD, STAD and UVM cancers. Its molecular function was closely associated with the C7orf70 and C7orf25 modules and the ribosomal pathway. Our study revealed the potential role of OPN3 in tumorigenesis and its prognostic value, suggesting that OPN3 might be a potential prognostic factor in these seven cancers.

## Supplementary Information

**Additional file 1: Figure S1.** The estimation of adjusted hazard ratios (HRs) and 95% confidence intervals (CIs) for survival outcomes using Cox regression model.

**Additional file 2: Figure S2.** Gene expression of OPN3 in different isocitrate dehydrogenase (IDH) mutations and grades of glioma in the CGGA dataset. Overall survival analysis of glioma patients between low and high expression of OPN3 groups in CGGA dataset according to OPN3 expression of median value using the Kaplan-Meier method.

**Additional file 3: Figure S3.** OPN3 expression of LIHC compared with adjacent normal tissues in the HCCDB dataset.

**Additional file 4: Figure S4.** OPN3 DNA methylation levels in five types of tumors compared to adjacent normal tissues. The Beta value indicates level of DNA methylation ranging from 0 (unmethylated) to 1 (fully methylated). Different beta value cut-off has been considered to indicate hyper-methylation [Beta value: 0.7 - 0.5] or hypo-methylation [Beta-value: 0.3 - 0.25] ^1^.

## Data Availability

The datasets used and/or analysed during the current study are available from the corresponding author on reasonable request.
