# Supplementary figures and images for: Integrated analysis of the prognostic and oncogenic roles of OPN3 in human cancers
Source: BMC Cancer. 2022 Feb 18;22:187. doi: 10.1186/s12885-022-09219-7 (PMC8857800; doi:10.1186/s12885-022-09219-7)

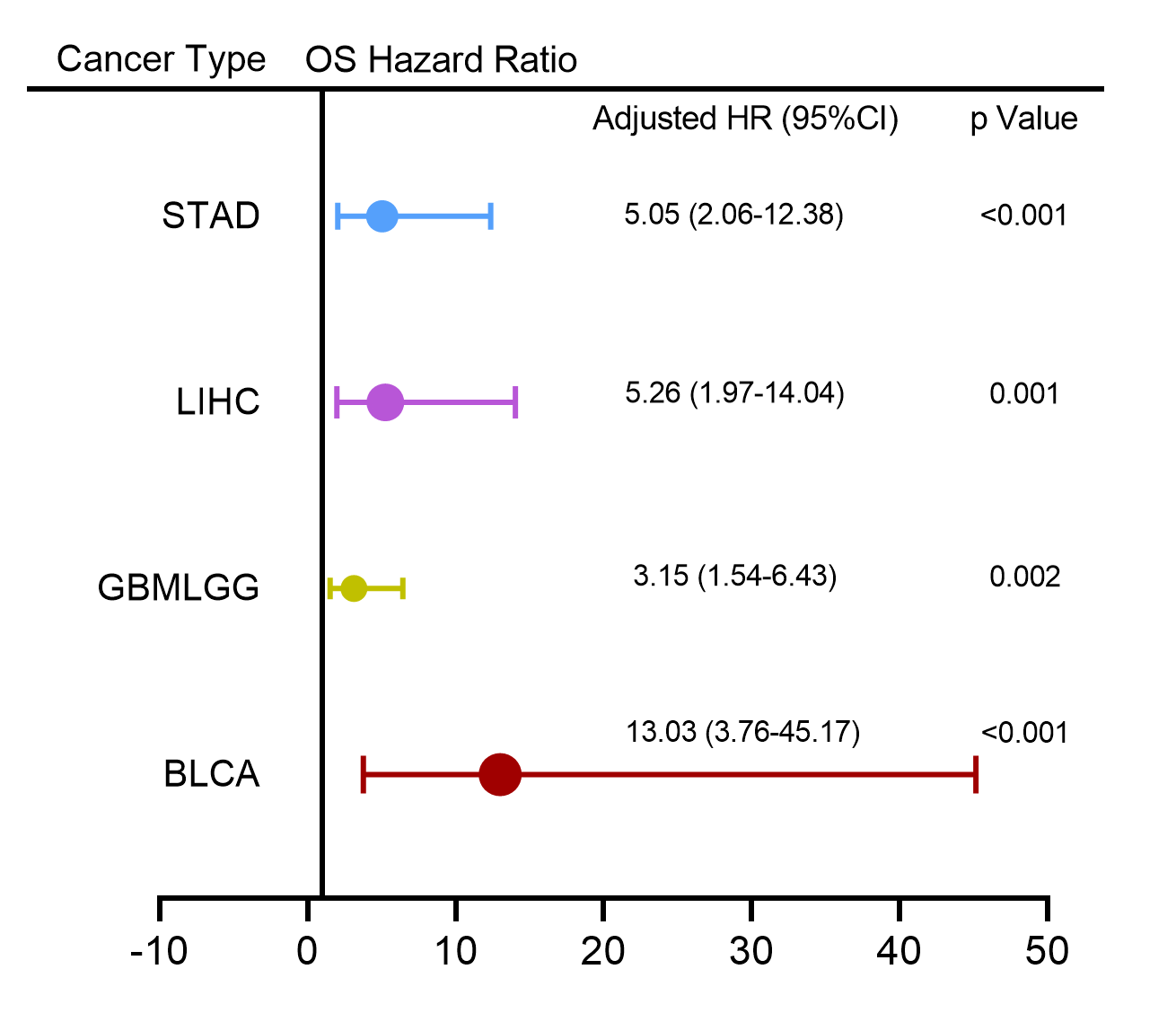

Supplement: Supplementary file 1 — Additional file 1: Figure S1. The estimation of adjusted hazard ratios (HRs) and 95% confidence intervals (CIs) for survival outcomes using Cox regression model. [file 12885_2022_9219_MOESM1_ESM.tif]

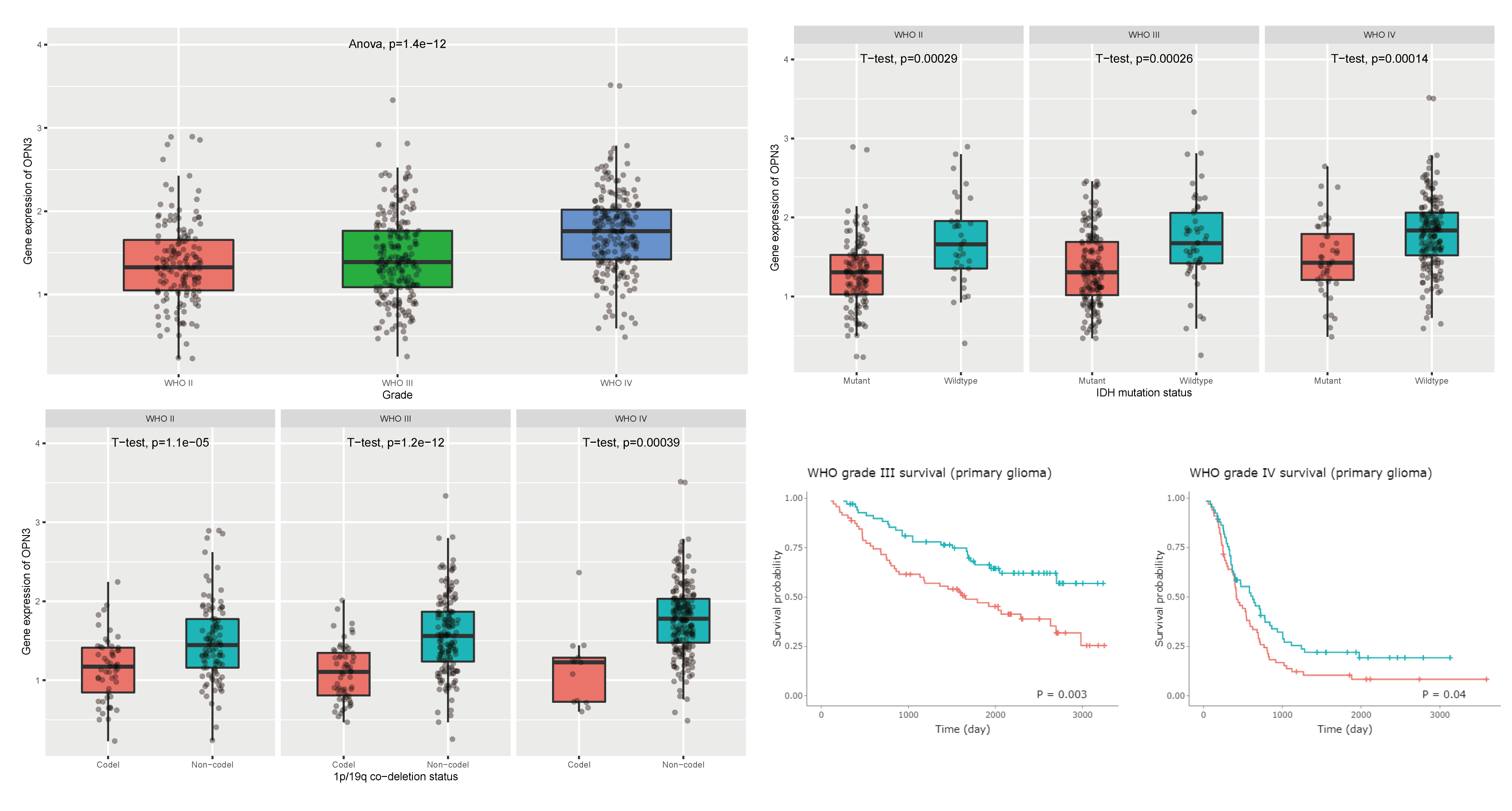

Supplement: Supplementary file 2 — Additional file 2: Figure S2. Gene expression of OPN3 in different isocitrate dehydrogenase (IDH) mutations and grades of glioma in the CGGA dataset. Overall survival analysis of glioma patients between low and high expression of OPN3 groups in CGGA dataset according to OPN3 expression of median value using the Kaplan-Meier method. [file 12885_2022_9219_MOESM2_ESM.tif]

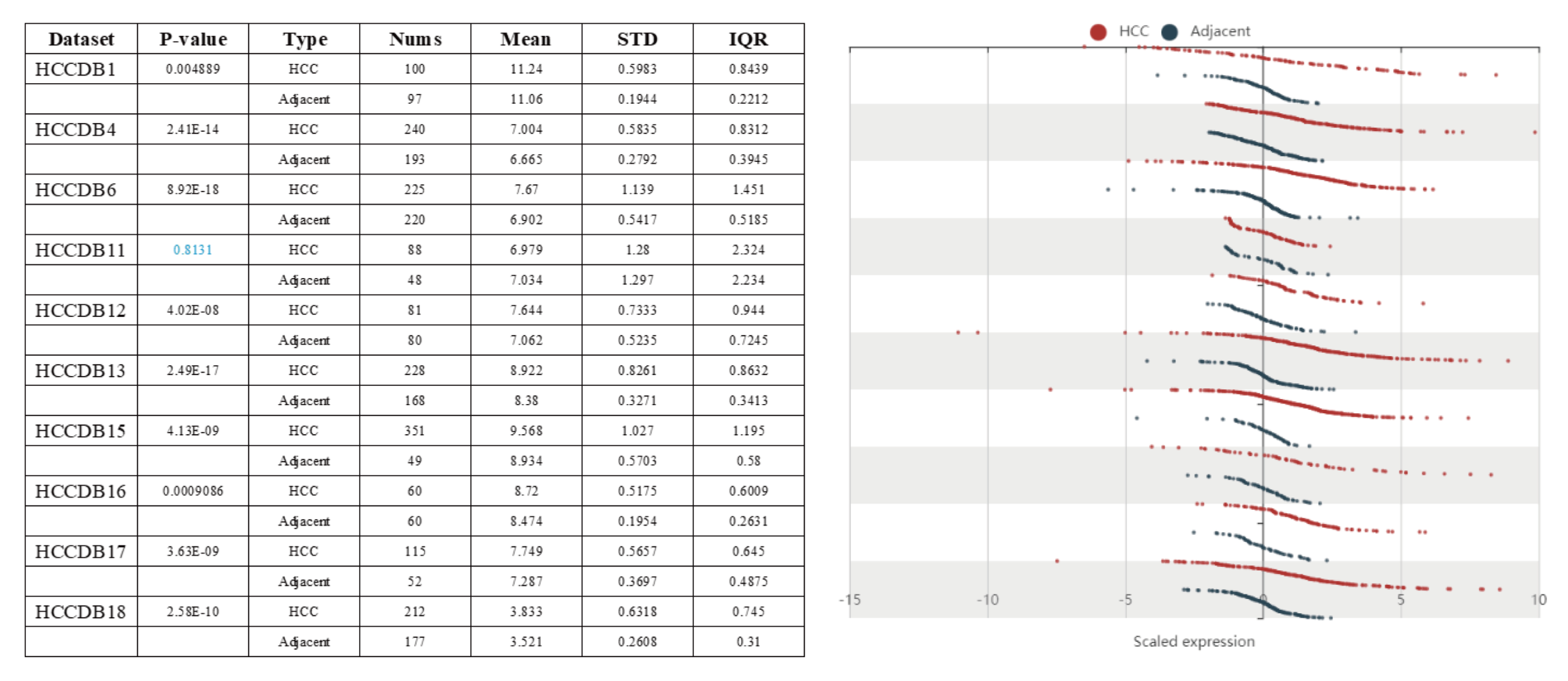

Supplement: Supplementary file 3 — Additional file 3: Figure S3. OPN3 expression of LIHC compared with adjacent normal tissues in the HCCDB dataset. [file 12885_2022_9219_MOESM3_ESM.tif]

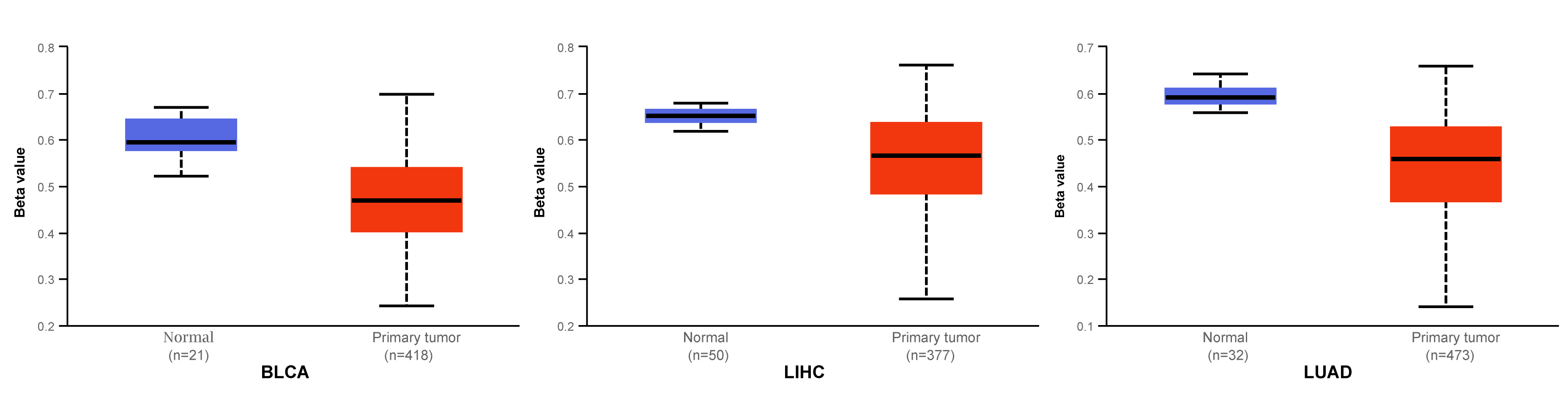

Supplement: Supplementary file 4 — Additional file 4: Figure S4. OPN3 DNA methylation levels in five types of tumors compared to adjacent normal tissues. The Beta value indicates level of DNA methylation ranging from 0 (unmethylated) to 1 (fully methylated). Different beta value cut-off has been considered to indicate hyper-methylation [Beta value: 0.7 - 0.5] or hypo-methylation [Beta-value: 0.3 - 0.25] 1. [file 12885_2022_9219_MOESM4_ESM.tif]
